# The impact of empirical superior vena cava isolation in addition to pulmonary vein isolation on outcomes in atrial fibrillation – Systematic review, meta-analysis, and meta-regression

**DOI:** 10.1016/j.ijcha.2025.101662

**Published:** 2025-03-25

**Authors:** Raymond Pranata, William Kamarullah, Giky Karwiky, Chaerul Achmad, Mohammad Iqbal

**Affiliations:** Department of Cardiology and Vascular Medicine, Faculty of Medicine, Universitas Padjadjaran, Hasan Sadikin General Hospital, Bandung, Indonesia

**Keywords:** Atrial fibrillation, Ablation, Superior vena cava isolation, Pulmonary vein isolation, Atrial tachyarrhythmia

## Abstract

**Background:**

Atrial tachyarrhythmia (ATa) recurrence remains high after pulmonary vein isolation (PVI), with the superior vena cava (SVC) being a common source of non-pulmonary vein foci.

**Objective:**

This *meta*-analysis aimed to synthesize the latest evidence and provide elaborate comparative analysis and *meta*-regression analysis regarding the empirical SVC isolation (eSVCi) versus no eSVCi in atrial fibrillation (AF) ablation.

**Methods:**

A systematic review was conducted on studies comparing eSVCi to no eSVCi (PVI-only or adjunctive SVC isolation). The primary outcome was ATa recurrence, defined as AF/atrial flutter/atrial tachycardia lasting over 30 s after a blanking period. Secondary outcomes included procedural duration, fluoroscopic duration, and complications.

**Results:**

Thirteen studies involving 2,176 patients were analyzed, with a mean follow-up of 18 ± 9.6 months. ATa recurrence was significantly lower with eSVCi (OR 0.54 [95 % CI: 0.41, 0.72], p < 0.001; I^2^ = 40.7 %). Subgroup analysis revealed eSVCi reduced ATa recurrence in observational studies but not in RCTs, and in initial ablations but not repeat procedures. eSVCi was beneficial in both PVI-only and adjunctive SVC isolation subgroups. Meta-regression showed the benefit of eSVCi was less in patients with non-paroxysmal AF (p = 0.044) and hypertension (p = 0.012). Procedural time, fluoroscopic time, and complications were similar between the two groups.

**Conclusion:**

eSVCi potentially reduce ATa recurrence compared to no eSVCi, without increasing complications or prolonging procedural times.

## Highlights

• Empirical superior vena cava isolation (eSVCi) was associated with lower ATa recurrence rates compared to non-eSVCi.
• eSVCi does not significantly prolong procedural or fluoroscopic durations.
• Complication rates were similar among eSVCi and non-eSVCi groups.
• The benefit was more pronounced in patients undergoing their initial procedure.
• The advantage of eSVCi appeared to be reduced in patients with non-paroxysmal AF and hypertension.

## 1 Introduction

The atrial tachyarrhythmia (ATa) recurrence following pulmonary vein isolation (PVI) in patients with atrial fibrillation (AF), particularly non-paroxysmal AF, remains high despite advancements in ablation strategies and technology. Although procedural efficiency has significantly improved with newer technologies, the recurrence rate has remained largely unchanged or only slightly improved. [1], [2] The role of additional ablation beyond PVI is still a matter of debate, but there is a clear need for supplementary strategies to further reduce ATa recurrence.

Non-pulmonary vein triggers are frequently implicated in AF, with the superior vena cava (SVC) being the most common source of non-pulmonary vein foci. [3], [4], [5], [6], [7], [8], [9], [10] These regions have garnered interest for ablation due to their arrhythmogenic potential. [11], [12], [13] Ectopic beats originating from the SVC are estimated to occur in about 33 % of cases, making it the most prevalent non-pulmonary vein source. [3], [8], [14], [15] However, the specific factors contributing to the SVC's arrhythmogenicity remain unclear. Empirical ablation of the SVC, without clear evidence of triggers or arrhythmogenicity, remains controversial. Previous *meta*-analyses based solely on randomized controlled trials demonstrated borderline statistical significance, and they did not explore which patient subgroups benefit most from this approach. [16], [17] This systematic review, *meta*-analysis, and *meta*-regression analysis aimed to synthesize the latest evidence and provide elaborate comparative analysis and *meta*-regression analysis regarding the empirical SVC isolation (eSVCi) versus no eSVCi in AF ablation.

## 2 Methods

### 2.1 Protocol and registration

This study was conducted in accordance with the Cochrane Handbook for Systematic Reviews of Interventions and reported based on the Preferred Reporting Items for Systematic Reviews and Meta-Analysis (PRISMA). The protocol was registered at the International Prospective Register of Systematic Reviews (PROSPERO), under identification number CRD42024599871.

### 2.2 Literature search strategy

**Fig. 1:**
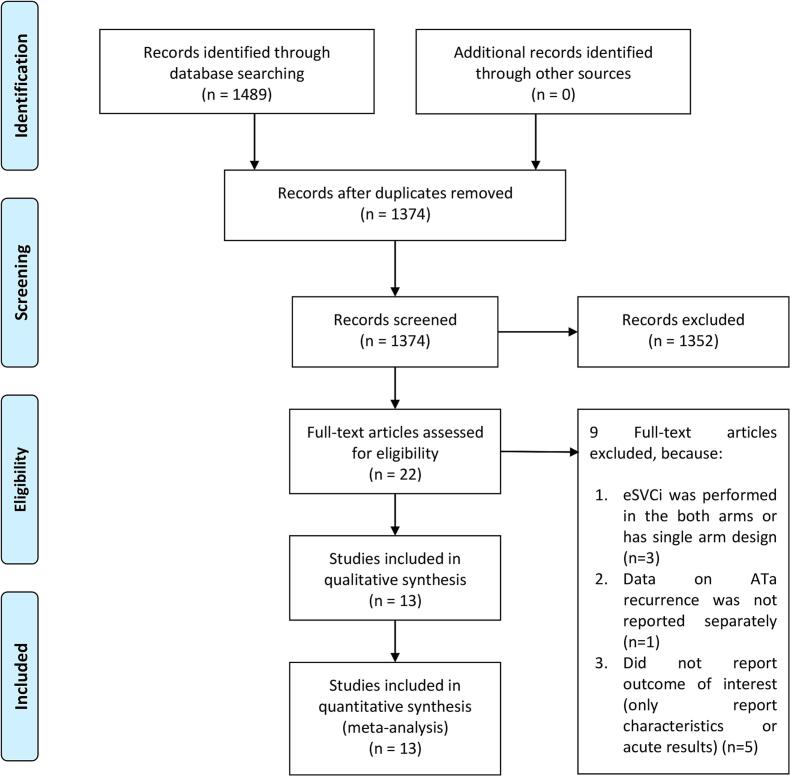
PRISMA flowchart. ATa: atrial tachyarrhythmia, eSVCI: empirical superior vena cava isolation.

We performed systematic literature search on PubMed, SCOPUS, and Europe PMC up to 7th October 2024. The search terms were as follows: (“Atrial Fibrillation” OR “AF” OR “atrial fibrillation”) AND (“Pulmonary Vein Isolation” OR “PVI” OR “pulmonary vein isolation” OR “pulmonary vein ablation”) AND (“Superior Vena Cava” OR “superior vena cava isolation” OR “SVC isolation” OR “SVCI” OR “superior vena cava ablation”) AND (“Catheter Ablation” OR “ablation” OR “AF ablation”). We tailored the search keywords to the requirements of each database. The literature search followed the PRISMA principles, the search and screening procedures is depicted in the Fig. 1.

### 2.3 Study Selection

We included randomized controlled trials and observational studies (both prospective and retrospective) reporting comparison between eSVCi to no eSVCi in terms of atrial tachyarrhythmia (ATa) recurrence. We excluded studies that did not provide a binary comparison of eSVCi versus no eSVCi, as well as additional papers, animal studies, editorials, comments, letters to editors, review articles, case reports/series, conference abstracts, and publications in languages other than English.

### 2.4 Intervention and control groups

The intervention group consisted of patients undergoing eSVCi in addition to PVI. eSVCi was defined as SVCi performed regardless of the presence of SVC triggers. The control group included patients who underwent a PVI-only procedure or those who received adjunctive SVCi, meaning SVCi was performed only if an SVC trigger was identified. This study aims to determine whether an empirical approach (prophylactically isolating the SVC without testing for triggers) is superior to isolating the SVC only when a trigger is identified or performing PVI alone without isolating the SVC. This will be assessed by pooling studies that compare patients who underwent SVCi regardless of the presence of SVC triggers with those who underwent PVI-only or SVCi only if a trigger was identified.

### 2.5 Outcomes of interests

The primary outcome of this study was ATa recurrence defined as atrial fibrillation/atrial flutter/atrial tachycardia (AF/AFL/AT) events lasting more than 30 s following ablation after blanking period. The secondary outcomes were the procedural duration, fluoroscopic duration, complications (especially non-vascular access related).

### 2.6 Data extraction and abstraction

The process of data extraction and abstraction was conducted by two independent authors with form detailing baseline characteristics of the included studies: sample size, study design, inclusion criteria, initial/repeat ablation, PVI or adjunctive SVCi in the control group, energy source, type of AF, male, age, diabetes, hypertension, left atrial (LA) diameter, left ventricular ejection fraction (LVEF), length of SVC sleeves, SVC trigger, follow-up length, and follow-up modality.

### 2.7 Risk of bias assessment

The risk of bias assessment was performed using the Cochrane risk of bias assessment tool for studies with randomized controlled trial (RCT) design. The Newcastle-Ottawa Scale (NOS) was used to assess risk of bias in observational studies, low risk of bias was defined as a total score of seven or above. The assessment was performed by two independent authors and discussion was held in the event of disagreements.

### 2.8 Statistical analysis

We used STATA 17 to perform statistical analysis in this systematic review and *meta*-analysis. The Hartung-Knapp-Sidik-Jonkman approach applies to both dichotomous and continuous data. The Hartung-Knapp-Sidik-Jonkman method yields more accurate error rates. [18] The effect estimate for binary comparison was odds ratio (OR). Estimation of heterogeneity was performed using the I-squared (I^2^) and a value of >50 % or P-value < 0.10 indicates statistically significant heterogeneity. To account for inter-study heterogeneity, random-effects model using Hartung-Knapp-Sidik-Jonkman method was used for the analyses, regardless of heterogeneity. A random effect restricted-maximum likelihood (REML) approach *meta*-regression analysis was also used to identify potentially significant modifiers for the primary outcome, curated from the characteristics of the included studies. A leave-one-out sensitivity analysis was performed to test for statistical robustness of pooled results. Subgroup analyses were performed for the study design, ablation energy used, initial or repeat ablation, and whether control group was adjunctive SVCi or PVI-only subgroups. Funnel plot analysis and Egger’s test was utilized to quantify the publication bias. Subsequent trim-and-fill analysis was performed due to asymmetrical funnel plot. All pooled analyses were two-tailed and were considered statistically significant if the p-value was below 0.05.

## 3 Results

Thirteen studies, including a total of 2,176 patients, were analyzed in this systematic review and *meta*-analysis [Fig. 1].[3], [15], [19], [20], [21], [22], [23], [24], [25], [26], [27], [28], [29] The baseline characteristics of the studies are provided in Table 1 and Table 2, and the studies were found to have a low risk of bias. The mean follow-up duration of the studies was 18 ± 9.6 months.

**Table 1:** Baseline characteristics of the studies.

Study	Sample	Design	Inclusion	Study Outcome	Initial/Repeat	Control	Non-paroxysmal AF	LAD (mm)	LVEF (%)	Length of SVC sleeves	SVC trigger (%)
Canpolat 2024	40 vs. 40 (80)	PSM PO and RO	Symptomatic Persistent AF undergoing CB PVI	AF/AFL/AT	Initial	PVI-only	100 vs. 100 (100)	43 vs. 44 (44)	59 vs. 60 (59)	NA	NA
Corrado 2010	134 vs. 160 (294)	RCT	Symptomatic AF refractory to AAD	AF	Initial	PVI-only	54 vs. 54 (54)	45 vs. 46 (46)	54 vs. 53 (54)	NA	3.1 (spontaneous)
Da Costa 2015	51 vs. 49 (100)	RCT	Symptomatic paroxysmal AF	AF/AFL/AT	Initial	PVI-only	0	42 vs. 39 (41)	63 vs. 64 (64)	NA	NA
Dong 2024	50 vs. 50 (100)	RCT	Symptomatic paroxysmal AF refractory to AAD	AF/AFL/AT	Initial	PVI-only	0	38 vs. 37 (38)	63 vs. 64 (63)	38 vs. 39 (39)	0
Ejima 2015	81 vs. 93 (174)	RO	Symptomatic paroxysmal AF	AF/AFL/AT	Initial	Adjunctive SVCi	0	36 vs. 36 (36)	56 vs. 55 (56)	NA	9 in control group
Gu 2022	29 vs. 30 (59)	RO	PVI non responders	AF/AFL/AT	First Repeat	Adjunctive SVCi	31 vs. 40 (36)	39 vs. 41 (40)	64 vs. 62 (63)	NA	7.8
Guan 2024	138 vs. 108 (246)	RO	Symptomatic paroxysmal AF	AF/AFL/AT	Initial	PVI-only	0	39 vs. 37 (38)	65 vs. 64 (65)	NA	NA
Knecht 2023	75 vs. 269 (344)	RO	Recurrent AF after index procedure	AF/Left AT	First Repeat	PVI-only	36 vs. 33 (34)	NA	58 vs. 59 (58)	NA	NA
Omuro 2021	102 vs. 51 (153)	RO	Non-paroxysmal AF	AF/AFL/AT	Initial	PVI-only	100	45 vs. 44 (45)	62 vs. 60 (61)	35 vs. 33 (34)	33 (arrhythmogenic SVC)
Overeinder 2020	50 vs. 50 (100)	RO	Paroxysmal AF	AF/AFL/AT	Initial	PVI-only	0	NA	NA	NA	4
Simu 2022	128 vs. 148 (276)	RO	Recurrent AF after ablation	AF	Repeat	PVI-only	31 vs. 33 (32)	26 vs. 27 (27)	55 vs. 54 (55)	NA	NA
Wang 2008	52 vs. 54 (106)	RCT	Paroxysmal AF refractory to AAD	AF/AFL/AT	Initial	PVI-only	0	37 vs. 36 (37)	62 vs. 62 (62)	NA	NA
Zhang 2020	72 vs. 72 (144)	RO	Recurrent paroxysmal AF after ablation	AF/AFL/AT	Repeat	Adjunctive SVCi	0	43 vs. 42 (42)	58 vs. 58 (58)	NA	NA

AF: atrial fibrillation, AFL: atrial flutter, AT: atrial tachycardia, AAD: anti-arrhythmic drugs, CB: cryoballoon, PO: prospective observational, RO: retrospective observational, PSM: propensity-score matched, PVI: pulmonary vein isolation, RCT: randomized controlled trial, SVC: superior vena cava, SVCi: superior vena cava isolation, LAD: left atrial diameter, LVEF: left ventricular ejection fraction, NA: not available.

**Table 2:** Baseline characteristics of the studies (continued).

Study	Energy Source	3D Mapping Systems and Other Related Catheters	RFA/CB Protocol	Ablation Line	Phrenic Nerve Injury Prevention	Male (%)	Age (mean, years)	Diabetes (%)	Hypertension (%)	Follow-up length (months)	Follow-up modality	NOS
Canpolat 2024	CB	Mapping catheter: Inner lumen circular mapping catheter (Achieve^TM^, Medtronic, Minneapolis, Minn, USA) CB catheter: Second generation 28 mm CB catheter (Arctic Front Advance^TM^, Medtronic, Minneapolis, Minn, USA)	The CB was inflated within the RA and positioned at the RA-SVC junction. The CB freeze lasted for 90 s. If SVCi was not achieved within 60 s, the CB was deflated and repositioned.	Circular or circumferential lesion	Pacing of right phrenic nerve from the SVC during freezing at the right-sided PVs with a 2000 ms cycle and a 12-mA output to detect phrenic nerve palsy	50 vs. 48 (49)	59 vs. 61 (60)	23 vs. 18 (21)	43 vs. 58 (50)	47	ECG and Holter	9
Corrado 2010	RFA	Mapping catheter: Circular mapping catheter (Lasso®; Biosense Webster, Diamond Bar, CA, USA) RFA catheter: an 8-mm RFA catheter (Celsius DS, Biosense Webster)	The circular mapping catheter was positioned above the junction of the RA and the SVC, aligned with the lower border of the pulmonary artery, under guidance from intracardiac echocardiography. RFA was conducted using a power of 50 W and a temperature of 60 °C.	Circular lesion	Output pacing (30 mA) at any site of the posterolateral side of the SVC In 13 % of patients planned for eSVCI, eSVCI was not performed due to risk of injury	74 vs. 74 (74)	55 vs. 57 (56)	NA	65 vs. 63 (64)	12	ECG and Holter	Low RoB*
Da Costa 2015	RFA	3D mapping system: CARTO®3 System Mapping catheter: Circular mapping (Navistar and Lasso) RFA catheter: A multipolar deflectable catheter (Lasso®; Biosense Webster, Diamond Bar, CA, USA)	RFA was conducted using an open irrigated-tip catheter, with the power output limited to a maximum of 25 W when applied to the posterior portion of the PV ostia or the SVC ostia.	Segmental ablation	Avoiding the posterolateral wall or high-output pacing stimulation (30 mA)	78 vs. 79 (79)	55 vs. 58 (56)	2 vs. 6 (4)	31 vs. 33 (32)	18	Holter	Low RoB*
Dong 2024	RFA	3D mapping system: CARTO®3 System RFA catheter: A 6 Fr decapolar catheter and two 6 Fr quadripolar catheters	The SVC-RA junction was identified as the horizontal line marking the ostium of the SVC. Segmental ablation was conducted at least 10 mm above the sinus node. RFA energy was applied at 40 W with a flow rate of 30 mL/min for the septal wall and at 35 W with a flow rate of 25 mL/min for the free wall.	Segmental ablation	Local maximum output pacing (20 mA) at the free wall of the SVC to tag the location of the phrenic nerve	70 vs. 74 (72)	59 vs. 57 (58)	12 vs. 8 (10)	50 vs. 48 (44)	12	Holter	Low RoB*
Ejima 2015	RFA	Mapping catheter: Circular mapping catheters (one duo-decapolar variable radius circular catheter [Lasso, Biosense-Webster, Baldwin Park, CA, USA], or Optima [St Jude Medical, Mineapolis, MN, USA]) RFA catheter: Navistar ThermoCool or ThermoCool SF, Biosense Webster Inc.	A circular mapping catheter was positioned 5–10 mm above the RA-SVC junction under the guidance of electroanatomic mapping. RFA energy was applied to the atrial tissue with a power range of 25–30 W. Irrigation rates were set at 17 mL/min when using the Navistar ThermoCool catheter and 8 mL/min with the Navistar ThermoCool SF catheter to ensure optimal power delivery.	Circumferential lesion	High output pacing (10 mA) at the posterior to lateral wall of the SVC	75 vs. 71 (73)	60 vs. 58 (59)	9 vs. 13 (11)	40 vs. 43 (42)	27	ECG & Holter	8
Gu 2022	RFA	3D mapping system: Carto, Ensite, or Rhythmia Mapping catheter: Pentaray, AFocus II, or Orion	Point-by-point ablation was performed 1–2 cm above the RA-SVC junction, with each point receiving 20–25 s of treatment. RFA was delivered at 20–30 W, using an open irrigation catheter with a saline irrigation rate of 17 ml/min and a maximum temperature of 42 °C.	NA	Pacing around the RA-SVC with an output of 20 mA to map the right phrenic nerve	66 vs. 50 (58)	62 vs. 60 (61)	4 vs. 23 (14)	38 vs. 57 (48)	20	ECG & Holter	8
Guan 2024	RFA	3D electro anatomical mapping system (CARTO 3, Biosense Webster) RFA catheter: ThermoCool SF, Biosense Webster Inc.	The SVCi procedure was conducted in accordance with AI-guided principles (values ranging from 350 to 400). RFA energy was delivered in a power-controlled mode with the following parameters: power set to 40 W, temperature maintained at 43 °C, saline irrigation at a rate of 15 mL/min, and contact force ranging between 5 and 20 g.	Integrated with ablation index (AI)	Point-by- point pacing with a contact force of 10–20 g along the phrenic nerve alignment.	70 vs. 63 (67)	61 vs. 60 (61)	25 vs. 22 (24)	54 vs. 58 (56)	16	Holter	8
Knecht 2023	RFA	3D electro anatomical mapping system (CARTO 3, Biosense Webster) Mapping catheter: Circular mapping (Lasso) RFA catheter: Navistar ThermoCool or ThermoCool SF, Biosense Webster Inc.	RFA energy was applied at the SVC using 25 W. Following PVI, the variable circular mapping catheter was withdrawn to the RA, and a detailed anatomical mapping of the SVC and its junction with the RA was conducted.	NA	Variable circular mapping catheter was placed 1 cm in the SVC. Pacing was performed using the ablation catheter with an output of 12 V and 2.9 ms to exclude local phrenic nerve capture Ablation was not performed or performed at 20 W in case of phrenic nerve capture	75 vs. 72 (73)	61 vs. 60 (60)	5 vs. 8 (7)	56 vs. 62 (61)	9	ECG & Holter	9
Omuro 2021	RFA	3D electro anatomical mapping system (CARTO 3, Biosense Webster) RFA catheter: Navistar ThermoCool SF, Biosense Webster Inc.	The geometry of the RA was reconstructed, and the SVC-RA junction was tagged on the geometry based on the SVC angiography. Segmental ablation targeting the earliest right atrium RA-SVC junction was conducted to address the SVCi, with contact force guidance of over 10 g. Irrigated RFA energy was applied for 20 s, maintaining a target temperature of 43 °C, a maximum power of 20–25 W, and an infusion rate of 17 mL/min. To minimize the risk of phrenic nerve injury, a lower energy setting (20 W) and reduced CF (10–15 g) were used on the lateral side.	Segmental ablation	High output pacing (10 mA) at the posterolateral aspect of the SVC Lower energy and contact force on the lateral site compared to septal site during ablation	73 vs. 89 (81)	66 vs. 61 (64)	22 vs. 17 (20)	61 vs. 50 (56)	18	ECG & Holter	9
Overeinder 2020	CB	Mapping catheter: Inner lumen circular mapping catheter (Achieve^TM^, Medtronic, Minneapolis, Minn, USA) CB catheter: Second generation 28 mm CB catheter (Arctic Front Advance^TM^, Medtronic, Minneapolis, Minn, USA)	To occlude the vessel, the cryoballoon was inflated in the right atrium and advanced toward the SVC ostium. Once total occlusion was confirmed through dye injection, showing complete retention of contrast in the SVC, cryoenergy application was initiated. A temperature limit of − 60 °C was maintained for the SVC ablation.	Circular or circumferential lesion	Pacing the ipsilateral phrenic nerve with a 1000-ms cycle and a 20-mA output	66 vs. 70 (68)	55 vs. 56 (56)	10 vs. 12 (11)	34 vs. 48 (41)	12	ECG & Holter	8
Simu 2022	RFA	3D electro anatomical mapping system (CARTO 3, Biosense Webster) Mapping catheter: Circular mapping catheters (One duo-decapolar variable radius circular catheter [Lasso, Biosense-Webster, Baldwin Park, CA, USA]. RFA catheter: ThermoCool SF, Biosense Webster Inc.	The SVCi procedure was performed following the guidance of AI values (350–450). RFA energy was applied with a power of 25 W for 10–15 s, maintaining a contact force of 10–25 g.	Integrated with ablation index (AI)	Pacing with maximal output (10 mV, 1 ms) on the lateral, anterolateral, and posterolateral regions of the SVC .	57 vs. 54 (55)	67 vs. 68 (67)	13 vs. 16 (15)	87 vs. 89 (88)	12	ECG & Holter	9
Wang 2008	RFA	Mapping catheter: Circular mapping catheters (One duo-decapolar variable radius circular catheter [Lasso, Biosense-Webster, Baldwin Park, CA, USA].	NA	Segmental or circumferential ablation	High output pacing (30 mA) on the postero-lateral wall of the SVC, then tagged on the geometry.	58 vs. 52 (55)	65 vs. 67 (66)	0 vs. 1 (1)	23 vs. 19 (21)	12	ECG & Holter	Low RoB*
Zhang 2020	RFA	Mapping catheter: The circular mapping catheter or 5-spline mapping catheter (Lasso or PenTarary, Biosense Webster, Irvine, CA) RFA catheter: A SmartTouch catheter (Biosense Webster, Irvine, CA)	RFA energy was delivered at 43 °C with a power of 25 W, and saline irrigation was administered at a rate of 17 ml/min. Segmental ablation was performed to target the earliest activation of SVC potential during sinus rhythm. Circumferential ablation, involving the continuous connection of ablation lesions to create a complete lesion line, was carried out during AF.	Segmental ablation	Output pacing (20 mA) was performed at the posterolateral wall of the SVC to tag phrenic nerve on the geometry	47 vs. 42 (45)	64 vs. 64 (64)	11 vs. 13 (12)	13 vs. 15 (14)	19	ECG & Holter	8

*Based on Cochrane risk of bias assessment tools for randomized controlled trials.

CB: cryoballoon, ECG: electrocardiography, RA: right atrium; RFA: radiofrequency ablation, RoB: risk of bias, NA: not available; PA: Pulmonary Artery; PVI: pulmonary vein isolation; SVC: superior vena cava; SVCi: superior vena cava isolation; W: Watt.

### 3.1 Atrial tachyarrhythmia recurrence

**Fig. 2:**
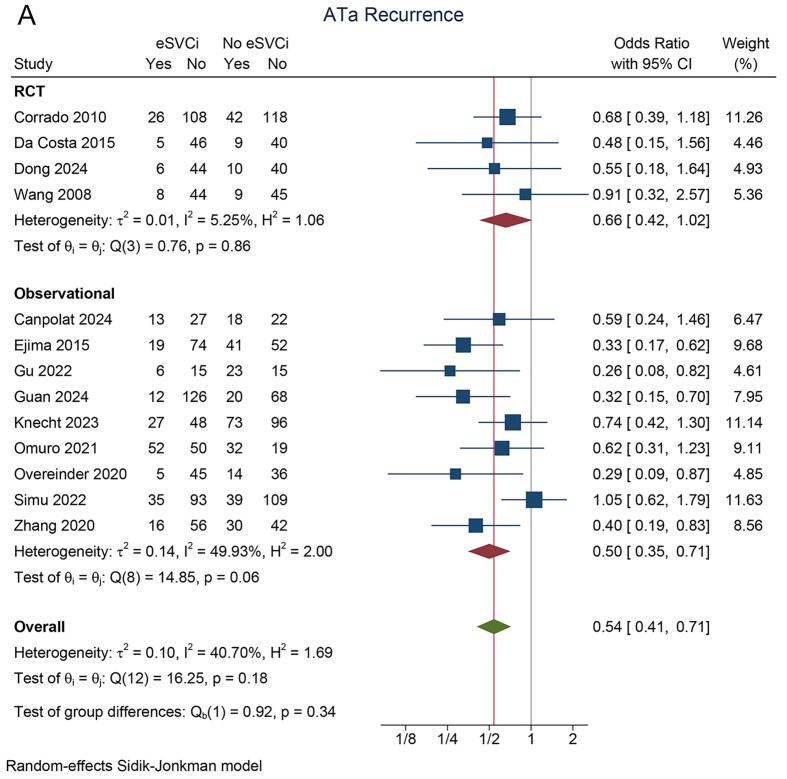

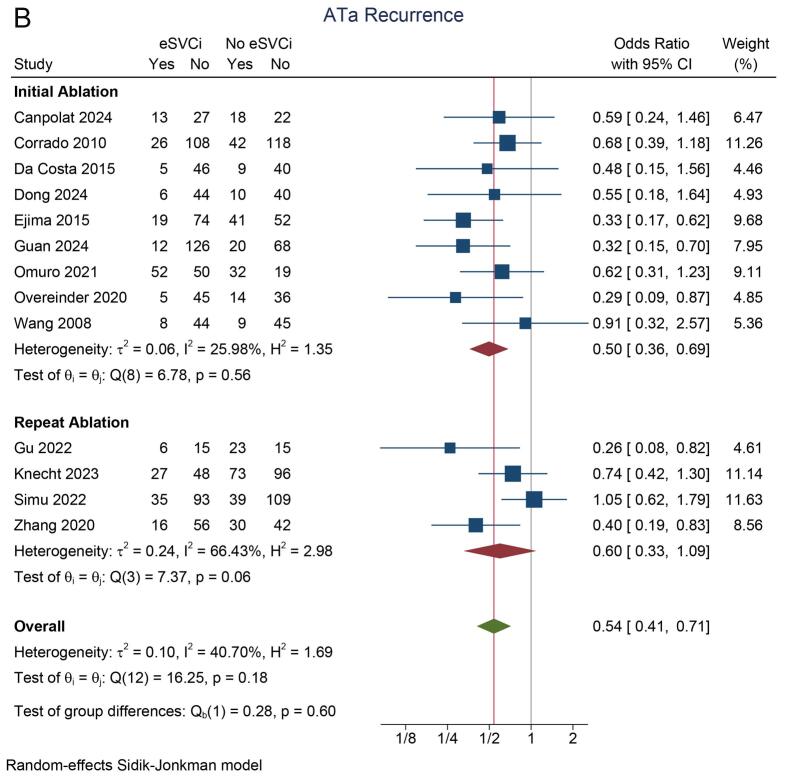

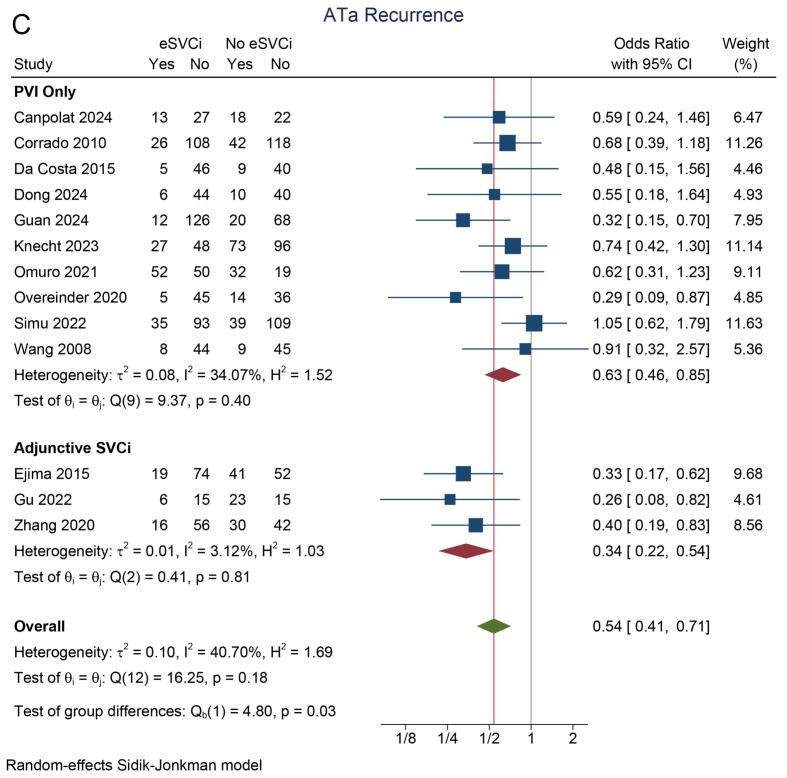

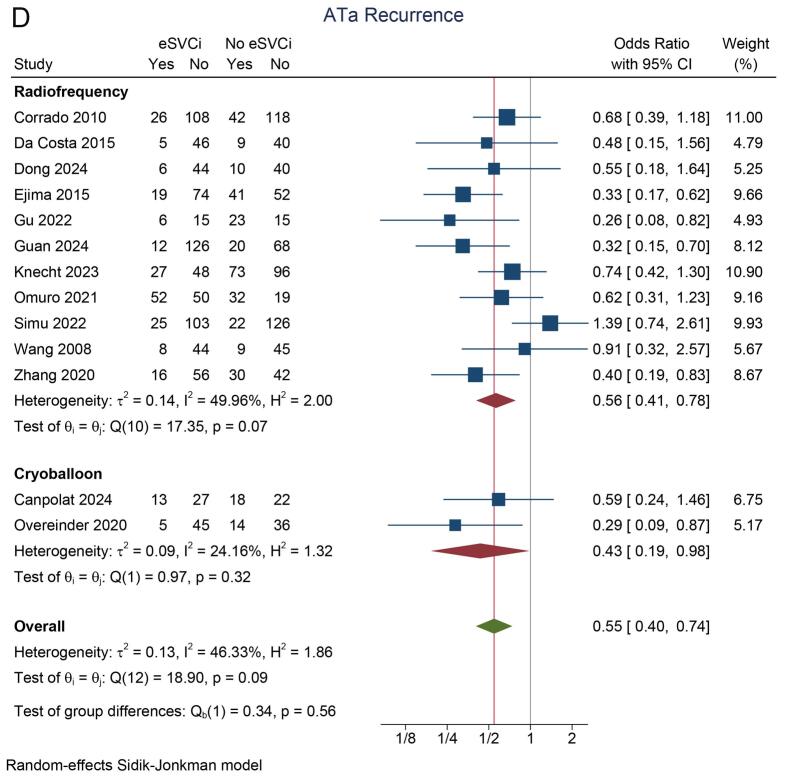
Atrial tachyarrhythmia recurrence. A) study design subgroup, B) initial/repeat ablation, C) adjunctive ablation or PVI-only, D) radiofrequency or cryoballoon energy source. ATa: atrial tachyarrhythmia, eSVCI: empirical superior vena cava isolation, PVI: pulmonary vein isolation, RCT: randomized controlled trial.

In the eSVCi group, the incidence of atrial tachyarrhythmia (ATa) recurrence was 22 % (95 % CI: 15 %, 29 %; I^2^ = 86.8 %). In contrast, the non-eSVCi group had an ATa recurrence rate of 34 % (95 % CI: 27 %, 42 %; I^2^ = 85.5 %). A pooled analysis demonstrated that ATa recurrence was significantly lower in patients who received eSVCi compared to those who did not (OR 0.54 [95 % CI: 0.41, 0.72], p < 0.001; I^2^ = 40.7 %, p_heterogeneity_ = 0.18) [Fig. 2A]. A leave-one-out sensitivity analysis consistently supported the benefit of eSVCi.

Subgroup analysis by study design showed that eSVCi significantly reduced ATa recurrence in observational studies (OR 0.50 [95 % CI: 0.35, 0.71]; I^2^ = 49.9 %) but not in RCTs (OR 0.66 [95 % CI: 0.42, 1.02]; I^2^ = 5.3 %) [Fig. 2A]. Another subgroup analysis showed that eSVCi significantly reduced ATa recurrence in patients undergoing initial ablation (OR 0.50 [95 % CI: 0.36, 0.69]; I^2^ = 26.0 %), but not in those undergoing repeat ablation (OR 0.60 [95 % CI: 0.33, 1.09]; I^2^ = 66.4 %) [Fig. 2B]. In both subgroups based on whether the control group had pulmonary vein isolation (PVI) only or adjunctive SVC isolation, eSVCi significantly reduced ATa recurrence (OR 0.63 [95 % CI: 0.46, 0.85]; I^2^ = 34.1 % and OR 0.34 [95 % CI: 0.22, 0.54]; I^2^ = 3.1 %) [Fig. 2C]. Additionally, subgroup analysis by ablation technique (radiofrequency or cryoballoon) showed significant reductions in ATa recurrence in both methods (OR 0.56 [95 % CI: 0.41, 0.78]; I^2^ = 50.0 % and OR 0.43 [95 % CI: 0.19, 0.98]; I^2^ = 24.2 %) [Fig. 2D].

Meta-regression analysis indicated that the benefit of eSVCi was lower in patients with non-paroxysmal AF (OR 1.01 [95 % CI: 1.00, 1.01] per % increase in population, R^2^ = 72 %, p = 0.044) and those with hypertension (OR 1.01 [95 % CI: 1.00, 1.02] per % increase in population, R^2^ = 100 %, p = 0.012). However, the benefit was not significantly influenced by age, sex (male), diabetes, left atrial diameter, left ventricular ejection fraction (LVEF), or follow-up duration (p > 0.05).

### 3.2 Procedural and fluoroscopic duration

**Fig. 3:**
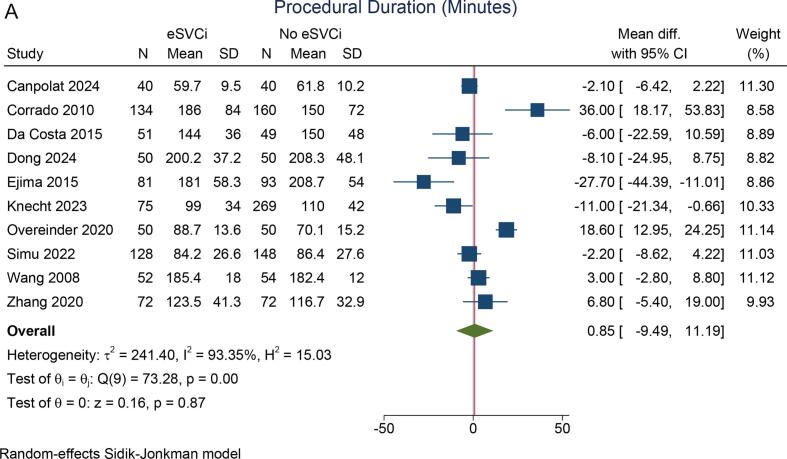

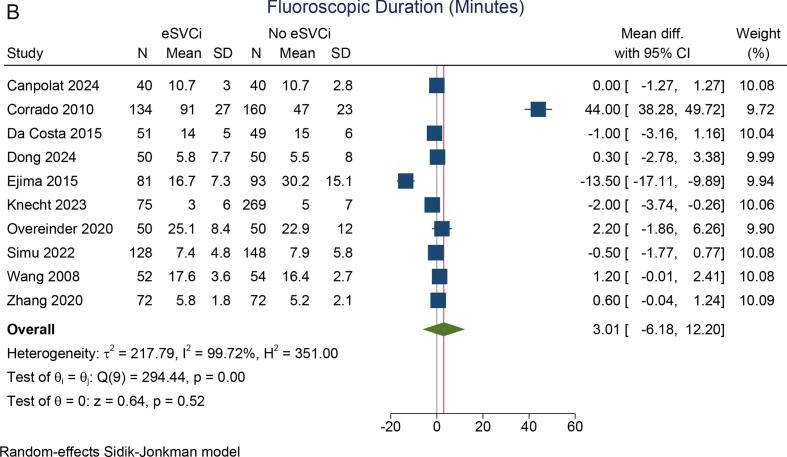
Procedural and fluoroscopic duration. A) procedural duration, B) fluoroscopic duration. eSVCI: empirical superior vena cava isolation.

The procedural duration did not differ significantly between the two groups (mean difference: 0.85 min [95 % CI: −9.49, 11.19], p = 0.872; I^2^ = 93.4 %, p_heterogeneity_ < 0.001) [Fig. 3A]. Similarly, the fluoroscopic duration was comparable (mean difference: 3.0 min [95 % CI: −6.18, 12.20], p = 0.520; I^2^ = 99.7 %, p_heterogeneity_ < 0.001) [Fig. 3B]. Both pooled analyses have high heterogeneity.

### 3.3 Complications

**Fig. 4:**
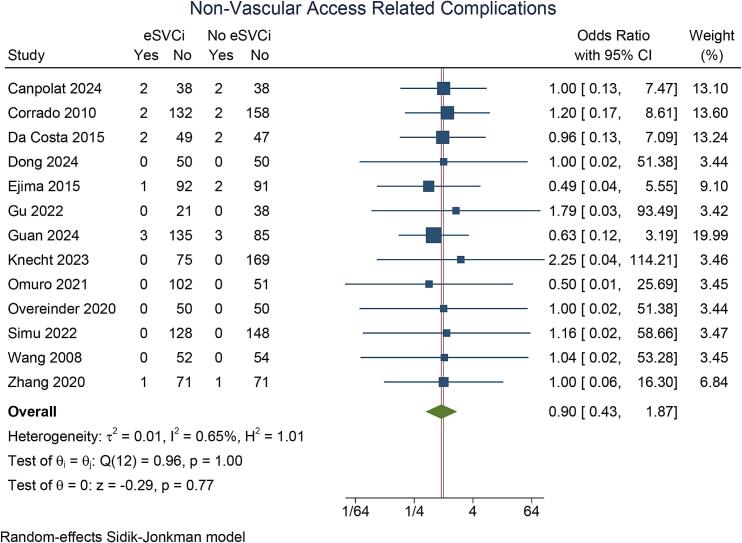
Non-vascular access related complications. eSVCI: empirical superior vena cava isolation.

Details on complications across the studies are presented in Table 3. Non-vascular access-related complications were similar between the eSVCi and non-eSVCi groups (OR 0.90 [95 % CI: 0.43, 1.87], p = 0.774; I^2^ = 0.7 %, p_heterogeneity_ = 1) [Fig. 4].

**Table 3:** Complications reported in the included studies.

Study	Complications
Canpolat 2024	SVCi: Femoral Hematoma 0 %, femoral pseudoaneurysm 2.5 %, right phrenic nerve palsy 5 % PVI: Femoral Hematoma 2.5 %, femoral pseudoaneurysm 0 %, right phrenic nerve palsy 5 %
Corrado 2010	SVCi: Tamponade 0.7 %, coronary artery embolism 0.7 % PVI: deep vein thrombosis 0.6 %, stroke 0.6 %, tamponade 0.6 %
Da Costa 2015	SVCi: transient phrenic nerve injury 1 %, phrenic nerve injury with partial recovery 1 % PVI: severe pulmonary stenosis 1 %, transient ischemic attack 1 %
Dong 2024	SVCi: None PVI: None
Ejima 2015	SVCi: gastric hypomotility 1.2 % Adjunctive SVCi: gastric hypomotility 1.1 %, cardiac tamponade 1.1 %
Gu 2022	SVCi: None Adjunctive SVCi: None
Guan 2024	SVCi: pericardial effusion 1.4 %, sinoatrial node injury 0.7 %, femoral pseudoaneurysm 1.4 % PVI: pericardial effusion 2.7 %, femoral pseudoaneurysm 0.9 %
Knecht 2023	SVCi: None PVI: None
Omuro 2021	SVCi: None PVI: Vascular access complications 2 %
Overeinder 2020	SVCi: None PVI: None
Simu 2022	SVCi: None PVI: None
Wang 2008	SVCi: Femoral pseudoaneurysm 3.8 % PVI: Femoral pseudoaneurysm 1.9 %
Zhang 2020	SVCi: Vascular access complications 2.8 %, transient phrenic nerve injury 1.4 % Adjunctive SVCi: Vascular access complications 7 %, transient ischemic attack 1.4 %

PVI: pulmonary vein isolation, SVC: superior vena cava, SVCi: superior vena cava isolation,

### 3.4 Publication bias

**Fig. 5:**
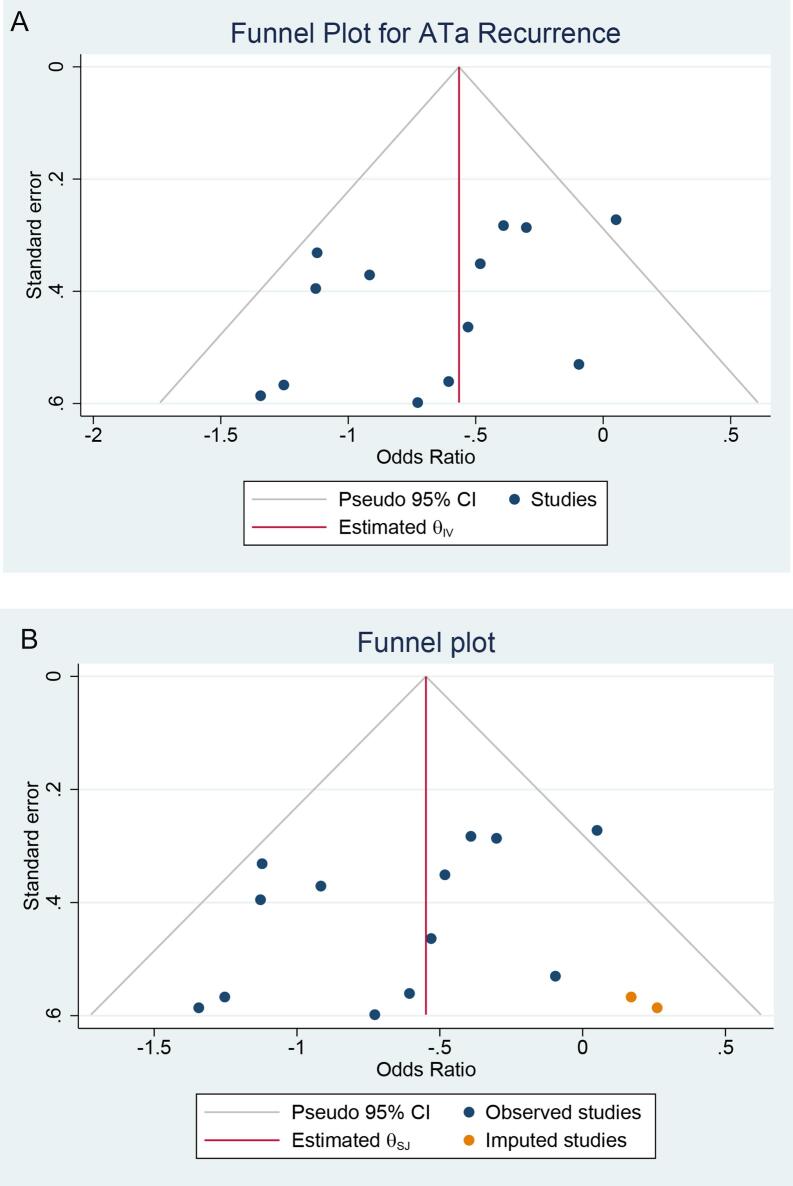
Publication bias. A) funnel-plot analysis, B) trim-and-fill analysis. ATa: atrial tachyarrhythmia.

Egger's test did not show significant small-study effects (p = 0.170) for ATa recurrence. The funnel plot exhibited slight asymmetry [Fig. 5A] with Simu 2022 et al. study being the outlier (upper right corner of the funnel plot), and trim-and-fill analysis imputed two studies to the right side of the plot [Fig. 5B], maintaining the significant benefit of eSVCi (OR 0.58 [95 % CI: 0.43, 0.77]).

## 4 Discussion

This *meta*-analysis demonstrated that eSVCi was associated with lower ATa recurrence rates compared to non-eSVCi, without significantly increasing procedural or fluoroscopic durations, and with similar complication rates. The benefit was more pronounced in patients undergoing their initial procedure compared to those having repeat ablation. However, the advantage of eSVCi appeared to be reduced in patients with non-paroxysmal AF and hypertension.

SVC is the most common non-pulmonary vein focus in AF, with arrhythmogenic SVC identified in 33 % of patients undergoing ablation. [3], [14], [15] The development of non-pulmonary vein foci, which often occurred in the SVC during follow-up, has been linked to ATa recurrence. [14] One study found that 22 % of patients undergoing repeat ablation were PVI non-responders, and non-pulmonary vein triggers were identified in 14.1 % of all patients, with 55 % of those located in the SVC. [26] Performing SVC provocation may be unnecessary due to the high prevalence of arrhythmogenic SVC, and the foci may also develop after ablation. A study indicated that the immaturity and limitations of provocative maneuvers may fail to unmask arrhythmogenic SVC or is not arrhythmogenic yet. [28] Subgroup analysis showed that compared to adjunctive SVCi, eSVCi significantly reduced the recurrence of ATa. Therefore, the prophylactic use of eSVCi seems to be a reasonable approach to reduce ATa recurrence. Studies have shown that SVC sleeves greater than 30 mm, SVC potentials exceeding 1 mV, and the distance from the top of the sinus node to the myocardial sleeve of the SVC are associated with more frequent SVC firing. [3], [11], [30], [31] However, whether patients with these characteristics would benefit more from eSVCi remains to be investigated.

Meta-regression analysis revealed that hypertension and non-paroxysmal AF were linked to a diminished benefit of eSVCi in AF patients. This may be due to these patients having more extensive left atrial fibrosis and substrate, where triggers play a less crucial role in sustaining non-paroxysmal AF. As a result, ablating both the pulmonary veins and the SVC may be insufficient for those with significant left atrial fibrosis.[32], [33], [34], [35] A similar issue might arise in patients undergoing repeat ablation, as subgroup analysis showed that eSVCi did not significantly reduce ATa recurrence in these patients. Since individuals with more extensive left atrial substrate are at higher risk for AF recurrence, repeat ablation patients may have AF triggers beyond the pulmonary veins and SVC.[36], [37], [38], [39] Despite this, the p-value was near statistical significance for benefit, suggesting that future studies could potentially alter the result.

In this *meta*-analysis, eSVCi had similar procedural durations compared to non-eSVCi. eSVCi can be performed during the observation period after PVI, meaning it does not significantly add to the procedure time. [25] By skipping the provocative maneuvers used in adjunctive SVCi, eSVCi saves time. [25] High heterogeneity was observed across studies regarding procedural durations, likely due to differences in energy sources, electrophysiology study, ablation protocol, and 3D mapping technologies. For example, cryoballoon ablation tends to have shorter procedure times compared to radiofrequency ablation. Cryoballoon ablation is a relatively new strategy for SVCi, with only two studies reporting its use. These studies demonstrated that cryoballoon ablation is both feasible and safe. The effect estimates from these studies are also closely aligned with the overall effect estimates. However, the efficacy of cryoballoon ablation compared to radiofrequency ablation for SVCi remains uncertain. Cryoballoon ablation may contribute to heterogeneity in the pooled effect estimates, previous studies suggested that the efficacy of radiofrequency and cryoballoon ablation were relatively mixed. [40], [41] Among radiofrequency ablations, the use of high-power short-duration techniques may also reduce procedural duration. [1] Previous studies have shown that SVCi using very high-power short-duration or high-power short-duration settings is feasible, effective, and safe, while also leading to shorter procedural durations. However, data on long-term outcomes remain limited. [42], [43].

Unfortunately, no studies compared eSVCi to non-eSVCi using PFA. As PFA becomes more widely adopted for AF treatment, there is increasing interest in whether additional ablation, such as eSVCi, is feasible and effective with the specific PFA catheter. [2], [4] A single-arm observational study and case report suggested that eSVCi with a pentaspline PFA catheter was feasible in all patients, with only transient complications that resolved by the end of the procedure and no sequelae at 3-month follow-up. [44], [45] However, more studies are needed to assess its effectiveness compared to no eSVCi.

Although there is concern that SVCi could lead to phrenic nerve or sinus node complications due to its proximity to the SVC, this *meta*-analysis found that non-vascular access related complications were rare, mostly transient, and did not significantly differ between the eSVCi and no eSVCi groups. The studies conducted extensive pacing in the SVC regions to trigger phrenic nerve stimulation and mapped the areas with positive responses. Subsequently, SVCi was performed by either avoiding these regions, reducing the applied power, or omitting the procedure entirely when a positive response was observed.

## 5 Limitations

One limitation is the varying lengths of follow-up across studies. However, *meta*-regression analysis indicated that the pooled effect estimates were not significantly influenced by follow-up length. There was a trend toward a reduction in ATa recurrence with eSVCi in the RCT subgroup with a borderline confidence interval indicating the need for more randomized trials, as the available trials may have been underpowered to detect statistically significant differences. However, since RCTs offer a much more robust level of evidence, the difference in the results of RCTs vs non-RCTs might be due to residual bias. Furthermore, the length of SVC sleeves and SVC potentials were reported in only a few studies, limiting analysis of whether specific patient characteristics related to the SVC might yield greater benefits from eSVCi. Atrial flutter and atrial tachycardia may not be directly attributable to SVC triggers. Therefore, AF recurrence could serve as a more specific outcome. However, most studies reported a composite outcome in the form of ATa. Finally, only one study, by Corrado et al., reported an aborted eSVCi due to the risk of injury (13 %). As a result, there is limited information on the feasibility of eSVCi and the reasons for its cancellation in general AF ablation populations.

## 6 Conclusion

eSVCi potentially resulted in lower rates of ATa recurrence compared to no eSVCi. It can be performed without significantly increasing procedural or fluoroscopic times, nor does it raise complication rates. The benefits of eSVCi seem to be more pronounced in patients undergoing their first ablation for paroxysmal AF.

Funding: None.

## CRediT authorship contribution statement

**Raymond Pranata:** Writing – review & editing, Writing – original draft, Methodology, Investigation, Formal analysis, Data curation, Conceptualization. **William Kamarullah:** Writing – original draft, Investigation, Formal analysis, Data curation. **Giky Karwiky:** Writing – review & editing, Investigation, Data curation. **Chaerul Achmad:** Writing – review & editing, Investigation, Data curation. **Mohammad Iqbal:** Writing – review & editing, Supervision, Methodology, Investigation, Formal analysis, Data curation, Conceptualization.

## Declaration of competing interest

The authors declare that they have no known competing financial interests or personal relationships that could have appeared to influence the work reported in this paper.
