# Supplementary material for: The impact of empirical superior vena cava isolation in addition to pulmonary vein isolation on outcomes in atrial fibrillation – Systematic review, meta-analysis, and meta-regression
Source: Int J Cardiol Heart Vasc. 2025 Mar 25;58:101662. doi: 10.1016/j.ijcha.2025.101662 (PMC11986545; doi:10.1016/j.ijcha.2025.101662)
Supplement: Supplementary Data 1 [file mmc1.docx]

**Supplementary Table 1. Newcastle Ottawa Scale**

| **Selection** | Canpolat 2024 | Corrado 2010 | Da Costa 2015 | Dong 2024 | Ejima 2015 | Gu 2022 | Guan 2024 | Knecht 2023 | Omuro 2021 | Overeinder 2020 | Simu 2022 | Wang 2008 | Zhang 2020 |
| --- | --- | --- | --- | --- | --- | --- | --- | --- | --- | --- | --- | --- | --- |
| Representative of the cohort? | * | * | * | * | * | * | * | * | * | * | * | * | * |
| Selection of the non-exposed | * | * | * | * | * | * | * | * | * | * | * | * | * |
| Ascertainment of exposure | * | * | * | * | * | * | * | * | * | * | * | * | * |
| Demonstration that outcome  was not present at start of study | * | * | * | * | * | * | * | * | * | * | * | * | * |
| **Comparability** |  |  |  |  |  |  |  |  |  |  |  |  |  |
| Comparability of cohorts on the basis of the design or analysis | * | * | * | * | * | * | ** | ** | * | ** | * | * | ** |
| **Outcome** |  |  |  |  |  |  |  |  |  |  |  |  |  |
| Assessment of outcome | * | * | * | * | * | * | * | * | * | * | * | * | * |
| Was follow up long enough for outcomes to occur | * | * | * | * | * | * | * | * | * | * | * | * | * |
| Adequacy of follow up of cohorts | * | * | * | * | * | * | * | * | * | * | * | * | * |
| Total quality score | 8 | 8 | 8 | 8 | 8 | 8 | 9 | 9 | 8 | 9 | 8 | 8 | 9 |
